# Association of Sun Safety Behaviors and Barriers with Sunburn History in College Students in a Region with High UV Exposure

**DOI:** 10.3390/curroncol29120759

**Published:** 2022-12-08

**Authors:** Dylan T. Miller, Zoe Baccam, Robin B. Harris

**Affiliations:** 1 Skin Cancer Institute at the University of Arizona Cancer Center, Tucson, AZ 85724, USA; 2 Mel and Enid Zuckerman College of Public Health, University of Arizona, 1295 N. Martin Ave., Tucson, AZ 85724, USA

**Keywords:** skin cancer, cancer prevention, risk factors, ultraviolet radiation (UVR)

## Abstract

Over five million cases of skin cancer are diagnosed each year in the United States with melanoma the third most common cancer in young adults. While publications have shown that sunburns increase the risk of developing melanoma throughout the lifetime including in adolescence and adulthood showing the importance of altering sun exposing behaviors throughout the lifetime, use of sun protection in college students remails low. In Fall 2019, an online survey of undergraduate students living on campus at a large southwestern university was conducted to determine the frequency of recent sunburns as well as sun protective behaviors and perceived knowledge of and barriers to sun protection. Associations between knowledge, behaviors, and barriers with self-reported sunburn were evaluated using logistic regression. Over 46% of 458 students reported at least one sunburn in the past three months and 21% reported having multiple sunburns in that period. Furthermore, 53% reported that they intentionally tanned their skin outdoors occasionally or more frequently, while 6.4% reported using an indoor tanning bed occasionally or more. Adjusted for skin sensitivity, recent sunburn history was associated with higher tanning activity scores and with high agreement that tanning was attractive (*p* < 0.01). This information can inform a more targeted series of intervention programming on the university campus.

## 1. Introduction

The incidence of both melanoma and nonmelanoma skin cancers has increased in the past decade [1]. It is estimated that nearly 5.4 million cases of skin cancer are diagnosed and treated in the United States every year, making skin cancer a burden both medically and financially [2,3]. Melanoma is the third most common cancer in young adults aged 15–39 [4]. Ultraviolet radiation (UVR) exposure is the number one modifiable risk factor for the development of melanoma, and excess sun exposure is estimated to cause as many as 90% of melanomas [5]. Publications have shown that number of sunburns increase the risk of developing melanoma throughout the lifetime, including sunburns in adolescence and adulthood [6,7] showing the importance of altering sun exposing behaviors throughout the lifetime. However, the literature continues to indicate that more than 50% of college students report a sunburn in the past year [8,9]. There is a need to further investigate UV exposure in this population and the behaviors, beliefs and barriers that are contributing to sunburn incidence. Past research has shown low levels of sun protection [8,9], low knowledge of sun safety [9,10], and high utilization of tanning activities [8,9,11]; however, these studies were not conducted in high UV locations.

We sought to determine college students’ use of sun-protective behaviors and the frequency of sunburns within the three months prior to the start of the semester at a large, southwestern US public university with high background UV levels throughout the year. Results of this research will be used to inform university policies and curricula to provide individuals with the information that they need to make informed, healthy choices about UVR exposure and promote policies that advance the national goal of preventing skin cancer, as laid out in the Surgeon General’s Call to Action to Prevent Skin Cancer [2]. 

## 2. Methods

### 2.1. Study Design

This cross-sectional survey was administered digitally through the Qualtrics software and conducted among undergraduate students living in dormitories at the University of Arizona in Tucson, Arizona. This survey was open for Fall semester of 2019 from 14 September to 3 November. Consent and survey completion were done online. The study was approved by the University of Arizona Institutional Review Board.

Recruitment was through emails sent out by resident assistants to their respective wings of a residence hall, professor emails to introductory level classes on campus, and posters with QR codes posted in residence halls. Recruitment materials included inclusion criteria: being a registered undergraduate student at the university. To increase recruitment efforts, incentives were for one student who finished the survey to be randomly selected to win an iPad. 

### 2.2. Survey Instrument

The survey was designed to determine students’ behaviors, knowledge, practices, and attitudes surrounding sun safety, skin cancer, and skin cancer prevention. The survey instrument was organized into 23 questions, including seven demographic questions, three knowledge questions, thirteen questions assessing sun-safe behaviors, and four questions assessing attitudes towards potential barriers to skin protection. Questions from this survey were adapted from surveys constructed by The Behavioral Measurement and Interventions Shared Resource at the University of Arizona Cancer Center, including questions adapted from the Arizona Skin Cancer Belief items, which were found to have acceptable internal consistency (Cronbach’s alpha = 0.67 in a middle school sample), and the Arizona Skin Cancer Self-Efficacy Items, which had acceptable internal consistency (Cronbach’s alpha= 0.78 in a middle school sample) [12].

Reported history of sunburn: The number of self-reported sunburns in the past three months were reported (0, 1, 2, 3, 4, 5+). Sunburn history also was recoded as a binary risk factor (0 vs. ≥1 sunburn). 

*Sun protection:* Sun-safe behaviors were assessed for five different behaviors. These behaviors included wearing a wide-brimmed hat, long sleeve shirt, sunglasses, use of sunscreen any time, and sunscreen on warm days. These behaviors were reported using Likert scale questions with 1 indicating low sun protection behavior and 5 indicating high sun protection behavior. Individual questions were described as well as a summary cumulative score across the five questions ranging from 5 to 25 (Sun Protection Score). 

Barriers to sun protection: Barriers to sun protection were assessed using five questions. These barriers included not being able to find shade on campus, sunscreen feeling bad on the skin, sunscreen making the skin break out, being tan making a person feel more attractive, and not having access to sunscreen. These behaviors were recorded using Likert scale questions with 1 indicating students reporting low belief in that item being a barrier to sun protection and 5 indicating the item was a high barrier to sun protection. Individual questions were described as well as a summary cumulative score across the five questions ranging from 5 to 25 (Barriers to Protection Score).

Perceived knowledge confidence about sun protection: Knowledge was assessed using three questions on the person’s confidence in knowing when to wear sunscreen, knowing how to protect the skin, and knowledge of the UV index. These behaviors were recorded using Likert scale questions with 1 indicating low confidence in their knowledge of sun protection and 5 indicating high confidence in their knowledge of sun protection. Individual questions were described as well as a summary cumulative score across the five questions ranging from 3 to 15 (Perceived Knowledge Confidence Score).

Intentional Tanning Activities: The high exposure activity score was comprised of two questions that included tanning bed use and intentional outdoor tanning. These activities were recorded using Likert scale questions with 1 indicating never participating and 5 indicating regular use. Individual questions were described as well as cumulative scores across the two questions with potential scores ranging from 2 to 10 (High Exposure Activity Score).

Participant characteristics and demographics: Demographic questions and skin characteristics included age, state or country of residence, gender (categories), race/ethnicity (categories), number of sunburns in the past three months, and skin sensitivity [12] Skin sensitivity was obtained in a six-point scale ranging from always burning and never tanning to never burning and tanning deeply [6]. 

### 2.3. Statistical Methods

Descriptive statistics were calculated for demographic and phenotypic characteristics, by binary sunburn occurrence. Chi-square tests were calculated to assess the association between demographical factors and binary sunburn occurrence. Mean and standard deviation of scores as well as the responses to each score’s component questions were also calculated. 

The magnitude of associations between recent sunburn history (0 versus ≥ 1 in the past 3 months) and students’ scores for perceived knowledge confidence, barriers to use of protection, use of protective methods, and participation in high exposure activities were evaluated using logistic regression to calculate odds ratios (OR) and 95% confidence intervals (CI). Odds ratios in the analyses for scores were interpreted as an increased odds per one unit increase in the independent risk factor. Crude and adjusted (AOR) models were estimated, with models adjusted for skin sensitivity (Types I–VI) Univariate logistic regressions were conducted to examine the association between each question included as a component of a score and sunburn history.

### 2.4. Sensitivity Analysis

A sensitivity analysis was conducted separately for males and females to measure the magnitude of associations between recent sunburn history and students’ scores for perceived knowledge confidence, barriers to use of protection, use of protective methods, and participation in high exposure activities. Logistic regressions were conducted to calculate odds ratios and 95% confidence intervals. Crude and adjusted (AOR) models were estimated, with models adjusted for skin sensitivity (Types I–VI).

## 3. Results

Of the 7000 students at the University of Arizona living in residence halls in August 2019, 530 surveys were completed. Participants with missing data for any knowledge, barrier, or protection question were dropped from the analysis resulting in 458 respondents. Responses from all 22 residence halls were represented. Table 1 presents demographic and personal characteristics, with the population predominately female (71.0%) and 2.4% identifying as non-cisgender. The age range was narrow as this study targeted students living in residence halls with 81.8% between the age of 18 and 20. The majority of individuals were white (52.3%) followed by multiracial (16.6%) and Hispanic or Latino (15.8%). The most common skin types were type III or IV skin (57.2%) with 17.5% reporting as Type I and II and 14.9% with Type V & VI skin types. While over half of the participants (53.1%) reported no sunburns in the past three months, 46.9% reported receiving at least one sunburn and 21.0% reported multiple sunburns and 7.9% reporting three or more sunburns. Over 55% of men reported a sunburn compared to 44% of women, however this difference was not statistically significant. Race and skin sensitivity types were statistically significantly associated with sunburn occurrence.

Table 2 displays the mean and standard deviation of each score as well as the distribution of responses to individual score component questions. The mean protection score was 13.66 and the mean barrier score was 13.48 out of a possible 25 points for each, which reflects low use of sun protection with low perception of barriers to use. The mean perceived knowledge score was 11.99 out of a possible 15 points which would indicate rather high perceived knowledge of how to protect their skin, while the mean intentional tanning score was 7.12 out of a possible 10 points. Only 27.5% of participants reported using sunscreen most of or all the time when in the sun. This frequency increased to 33.2% for utilizing sunscreen on warm days. Use of sun-protective clothing also was not prevalent with 10.3% reporting that they wear a wide-brimmed hat all or most of the time when in the sun and 11.8% utilizing a long-sleeved shirt, although 50.3% reported wearing sunglasses all or most of the time. 

For barriers to sun protection, 88.7% of participants agreed that they had access to sunscreen, and 62% agreed that they could find shade on campus. However, 62.9% of these undergraduates reported that being tanned made them feel attractive. Overall, participants reported high confidence in their knowledge of sun protection. More than 80% strongly or somewhat agreed that they have the knowledge to protect their skin from the sun and had the knowledge to select an appropriate sunscreen.

Most of the participants participated in outdoor activities (82.3%) and 53.3% reported that they intentionally tanned their skin outdoors occasionally or more frequently. A smaller percentage (6.4%) of individuals reported using an indoor tanning bed occasionally or more. 

Table 3 summarizes the magnitude of the associations of the sun protection behaviors, barriers, perceived knowledge, and tanning activity scores and the individual score components. Increased Sun Protection Score was statistically significantly associated with sunburn occurrence before adjustment, however after adjustment f for skin sensitivity type, it was not statistically significant. Similarly for specific types of sun protection, the unadjusted univariate models for use of sunscreen and wearing a wide-brimmed hat were associated with sunburn occurrence but not after adjustment. Only use of wide-brimmed hats remained statistically significant in the adjusted model. 

The Barriers to Protection Score was not significantly associated with sunburn occurrence in either the crude or adjusted models. However, the individual component focused on perception that a tan makes one more attractive was associated with sunburn occurrence in both the crude (OR 1.33, 95% CI 1.13, 1.16) and adjusted models (AOR 1.36, 95% CI 1.14, 1.61).

The Perceived Knowledge Confidence Score was not significantly associated with sunburn occurrence in this population in either model (AOR 1.06, 95% CI 0.98, 1.16), although students’ perceived knowledge that they knew the UV index was associated with sunburns (AOR 1.19, 95% CI 1.02, 1.39).

The overall High Exposure Activity (or tanning) Score was significantly associated with sunburn history even after adjustment (AOR 1.33, 95% CI 1.16, 1.52). Furthermore, both separate activities were statistically significant. 

In a sensitivity analysis that looked for potential differences between genders in the associations between sunburn history and specific behaviors (Table 4), AORs for protection scores were statistically significant for males but not for females. Likewise, self-reported sunscreen use was significantly associated with an increase in sunburn occurrence for males but not for females. However, associations with a sunburn making one feel attractive were statistically significant for females, although the odds ratios were similar between the two groups. For perceived knowledge, the knowledge to check the UV index was statistically significant for females but was not for males. Finally, excessive exposure scores remained s for both males and females; however, indoor tanning use became insignificant for both genders.

## 4. Discussion

This study of college students in Arizona, a geographical area with one of the highest mean UV indices in the United States [13], shows that 47% of students reported receiving at least one sunburn in the last 3 months and 21% reported multiple sunburns. A number of other studies show that college students are receiving an alarming number of sunburns throughout the year, reporting that between 55–65% of college students receive a sunburn each year [8,9]. These studies, however, were conducted in regions with lower average UVR, namely the northeastern United States. While the current study percentage is less than that reported in other universities, this percentage still is excessive. 

Levels of use of sun protective behaviors were low in this population, although those reporting higher protective usage were more likely to report receiving a sunburn. These associations were not statistically significant, however, after adjustment for skin type. Interestingly, the association of the use of wide-brimmed hats and sunburns remained statistically significant. 

Similar associations have been seen in older adults [14] and adolescents [15], which may be due to the use of sunscreen by those who are at high risk of being sunburned. The association of wide-brimmed hat and continued sunburning may be due to hat type selected or dependence upon only one sun protection behavior. Location of the sunburn was not identified in the current survey. Past literature supports the lack of regular sun protection among college students with studies showing between 15–41% utilizing sunscreen sometimes, always or often, 46–45% wearing wide-brimmed hats, and 21–72% wearing long-sleeved shirts [8,9]. Protective methods were higher in one study located in a cloudy environment with 66% utilizing sunscreen sometimes or more, 82% wearing sunglasses, and 80% wearing long-sleeved shirts [11]. Sun protective behaviors may be an appropriate avenue to address the high levels of sunburn in this population as a systematic review of school-based sun safety interventions found that most interventions improved sun protection behaviors but few were able to reduce the time students were spending in the sun [16].

Identification of barriers to sun protection were generally low in this population. This finding aligns with past research which shows low self-reported barriers to protection including the feel of sunscreen [17,18]. The belief that a tan makes a person feel more attractive was the most reported barrier to sun protection and was associated with sunburn. This finding aligns with past research which has shown that the belief that a tan is attractive is significantly associated with the perceived importance of tanning [19]. It has also been shown that a belief that tanning improves appearance is significantly associated with lower likelihood of having “high” sun protective behaviors [19]. 

Perceived knowledge of sun protective methods was high in this study, although this could have been due to confirmation bias. Certainly, one prior study indicated that a high level of skin cancer prevention knowledge is not related to a reduction in the perceived importance of tanning [11]. Additionally, prior studies that have used questionnaires to assess the knowledge of college students have had mixed results. One study found that only 22.5% of college students could answer sunscreen knowledge questions correctly [9], while another found that over 85% of college students had “satisfactory” knowledge of sunscreen and sun safety [10]. The findings of this study suggest that perceived knowledge of sun safety methods is not related to practice of sun protection and may not directly impact the likelihood of receiving a sunburn in college students. 

Findings from this study support previous literature showing a high level of intentional outdoor tanning among college students. Over 53% of these college students said the occasionally, often, or regularly intentionally tan. These findings from Arizona support the prior research that also showed over half of college students engaged in intentional outdoor tanning [8,9,11]. While intent to tan is relatively similar between the studies, the reported sunburn history is somewhat lower in Arizona. Potentially the lower UV exposure regions are associated with lower sun exposure throughout the year with students in this region more susceptible to intermittent sun exposure causing sunburns in the spring and summer. Less than ten percent of individuals in this population utilized tanning beds which is aligned with some past research [8,9]. The low utilization of tanning beds may also be due to regional differences as past literature has shown that colleges in the western United States have lower access to tanning beds on campus than colleges in other regions [20]. However, a targeted approach may be needed to identify groups who frequently use tanning beds on campus. One study among white college students who were fraternity or sorority members found that 58% of males and 99% of females had ever used a sunlamp, tanning lamp, or sunbed [19]. Preventing the use of tanning beds is vital to long term health as excessive indoor tanning appears to show signs of behavioral addiction such as difficulty quitting and excessive motivation and urges to tan [21].

Limitations of this study include the use of a cross-sectional survey with self-reported characteristics and perceptions and probable recall bias. The finding that 17–20% of individuals with skin types V and VI (or rarely or never burn) reporting sunburns in the past three months may suggest an issue with overreporting. However, sunburn history was substantially higher for skin types I–IV. The use of perceived knowledge instead of testing true knowledge of sun protection could lead to an overestimation of what the students’ truly know. Perceived knowledge of sun safety as a proxy for true knowledge has not been tested for reliability or validity. In addition, this study was conducted in a high UV region with students reporting sunburns that occurred during the summer months and may not be generalizable to other regions of the United States. However, the sunburn frequency reported by these students is similar or even higher than reported in other geographic locations with lower UV indices. Strengths of this study include relatively large sample size and the use of skin type in analysis instead of race as a proxy for skin type. This may improve the validity of this study compared to past literature. 

## 5. Conclusions

College students at a large public university in Arizona continue to receive an alarming number of sunburns and further interventions must be developed to target this population. The disconnect between perceived knowledge and action practices need to be addressed. Future interventions must also address the perception among college students that a tan is attractive and the high levels of intentional tanning activities. 

## Figures and Tables

**Table 1:** Demographic and personal characteristics of college students by history of self-reported sunburn in the past three months.

Characteristics	1 + Sunburn in Past 3 Months *N* (%)	No Sunburns in Past 3 Months *N* (%)	*p*-Value *
Race/Ethnicity			
White	147 (61.51)	92 (38.49)	<0.01
African American or Black	2 (12.50)	14 (87.50)	
Multiracial	19 (25.00)	57 (75.00)	
Hispanic or Latino	29 (40.28)	43 (59.72)	
Asian or Asian American	9 (24.32)	28 (75.68)	
Other	8 (47.06)	9 (52.94)	
Gender			
Woman	145 (44.62)	180 (55.38)	0.14
Man	65 (55.08)	53 (44.92)	
Other	5 (33.33)	10 (66.67)	
Skin Type			
Always burns, never tans (Type I)	31 (67.39)	15 (32.61)	<0.01
Usually burns, tans minimally (Type II)	53 (65.43)	28 (34.57)	
Sometimes burns, tans (Type III)	87 (54.72)	72 (45.28)	
Rarely burn, tans well (Type IV)	31 (30.39)	71 (69.61)	
Rarely burn, tans deeply (Type V)	7 (20.59)	27 (79.41)	
Never burns, tans with extreme exposure (Type VI)	6 (17.65)	28 (82.35)	

* *p* value for Chi Square test.

**Table 2:** Overall sun protection behaviors, knowledge, and barriers scores and individual component question responses among college students, 2019.

Question	Never *N* (%)	Almost Never *N* (%)	Occasionally *N* (%)	Most of the Time *N* (%)	Always/Almost Every Day *N* (%)
Use of Sun Protection					
Overall score: Mean, SD	13.66, 3.5				
Wear sunscreen on warm days	34 (7.42)	138 (30.13)	134 (29.26)	88 (19.21)	64 (13.97)
Wear sunscreen	22 (4.80)	128 (27.95)	182 (39.74)	70 (15.28)	56 (12.23)
Wear long-sleeved shirt	128 (27.95)	144 (31.44)	132 (28.82)	40 (8.73)	14 (3.06)
Wear sunglasses	53 (11.57)	58 (12.66)	117 (25.55)	104 (22.71)	126 (27.51)
Wear wide-brimmed hat	217 (47.38)	116 (25.33)	78 (17.03)	39 (8.52)	8 (1.75)
Intentional Tanning					
Overall score: Mean, SD	7.12, 2.04				
Use a tanning bed	413 (90.57)	14 (3.07)	18 (3.95)	6 (1.32)	5 (1.10)
Intentionally tan outdoors	127 (27.73)	87 (19.00)	133 (29.04)	77 (16.81)	34 (7.42)
Perceived Knowledge					
	Strongly Disagree	Somewhat disagree	Neither agree nor disagree	Somewhat agree	Strongly agree
Overall Score: Mean, SD	11.99, 2.3				
I have the knowledge to protect my skin	4 (0.87)	14 (3.06)	33 (7.21)	207 (45.20)	200 (43.67)
I have the knowledge to select sunscreen	8 (1.75)	24 (5.24)	47 (10.26)	183 (39.96)	196 (42.79)
I have the knowledge to check and interpret the UV index	37 (8.08)	84 (18.34)	62 (13.54)	144 (31.44)	131 (28.60)
Barriers to Use of Protection					
Overall Score: Mean, SD	13.48, 3.01				
Sunscreen makes my skin break out	102 (22.27)	102 (22.27)	86 (18.78)	112 (24.45)	56 (12.23)
I can’t find shade on campus	91 (19.87)	193 (42.14)	59 (12.88)	100 (21.83)	15 (3.28)
Sunscreen feels bad on my skin	73 (15.94)	136 (29.69)	89 (19.43)	105 (22.93)	55 (12.01)
I don’t have access to sunscreen	283 (61.79)	123 (26.86)	20 (4.37)	24 (5.24)	8 (1.75)
A tan makes me feel attractive	34 (7.42)	29 (6.33)	107 (23.36)	132 (28.82)	156 (34.06)

**Table 3:** Association of self- reported use of sun safety for protection, barriers, perceived knowledge of sun safety, and tanning history with recent history of sunburn among college students, 2019.

Scores and Component Questions	Crude OR *	95% CI	*p*-Value	Adjusted AOR *	95% CI	*p*-Value
Use of Sun Protection						
Overall Protection Score	1.07	1.02, 1.13	0.01	1.05	0.99, 1.11	0.12
Sunscreen	1.22	1.03, 1.46	0.03	1.15	0.95, 1.39	0.15
Sunscreen on warm days	1.12	0.96, 1.31	0.16	1.05	0.88, 1.24	0.60
Long-sleeved shirt	1.07	0.90, 1.28	0.78	0.99	0.83, 1.20	0.99
Wide-brimmed hat	1.26	1.06, 1.50	0.01	1.22	1.02, 1.47	0.03
Sunglasses	1.08	0.94, 1.24	0.29	1.06	0.91, 1.23	0.45
Barriers to Use of Sun Protection						
Overall Barriers Score	1.04	0.98, 1.11	0.21	1.03	0.97, 1.10	0.31
Sunscreen makes my skin break out	1.06	0.93, 1.22	0.39	0.99	0.86, 1.15	0.92
I can’t find shade on campus	0.87	0.74, 1.03	0.1	0.92	0.77, 1.09	0.32
Sunscreen feels bad on my skin	1.00	0.87, 1.16	0.97	0.96	0.82, 1.12	0.60
I don’t have access to sunscreen	1.03	0.84, 1.25	0.79	1.10	0.89, 1.37	0.38
A tan makes me feel attractive	1.33	1.13, 1.56	<0.01	1.36	1.14, 1.61	<0.01
Perceived Knowledge of Sun Safety						
Overall Knowledge Score	1.06	0.98, 1.16	0.15	1.06	0.97, 1.15	0.19
Knowledge to protect the skin	1.14	0.90, 1.44	0.27	1.14	0.89, 1.46	0.29
Knowledge to select appropriate sunscreen	0.96	0.79, 1.17	0.68	0.93	0.75, 1.15	0.49
Knowledge to check and interpret the UV index	1.18	1.02, 1.36	0.03	1.19	1.02, 1.39	0.03
Intentional Tanning						
Overall Tanning Score	1.31	1.16, 1.49	<0.01	1.33	1.16, 1.52	<0.01
Tanning bed use	1.42	1.05, 1.92	0.02	1.53	1.12, 2.10	0.01
Intentional outdoor tanning	1.37	1.18, 1.60	<0.01	1.39	1.18, 1.63	<0.01

* ORs interpreted as change for each level of response. AOR adjusted for skin sensitivity (6-point scale).

**Table 4:** Association of self-reported use of sun safety, perception of knowledge of sun safety, and barriers with recent history of sunburn among male and female college students, 2019.

	Male			Female		
Scores and Component Parts	Adjusted OR *	95% CI	*p*-Value	Adjusted OR	95% CI	*p*-Value
Use of Sun Protection						
Overall Protection Score^c^	1.14	1.01–1.29	0.04	1.02	0.95–1.09	0.55
Sunscreen	1.99	1.23–3.22	0.01	1.13	0.94–1.37	0.19
Sunscreen on warm days	1.28	0.88–1.88	0.2	1.02	0.83–1.25	0.84
Long-sleeved shirt	1.16	0.82–1.64	0.39	0.92	0.73–1.16	0.47
Wide-brimmed hat	1.26	0.89–1.78	0.19	1.21	0.95–1.53	0.12
Sunglasses	1.09	0.81–1.46	0.57	1.04	0.86–1.24	0.7
Barriers to Use of Sun Protection						
Overall Score	0.99	0.87–1.14	0.94	1.06	0.98–1.14	0.15
Sunscreen makes my skin break out	1.00	0.76–1.31	0.98	0.99	0.83–1.19	0.92
I can’t find shade on campus	0.83	0.59–1.18	0.3	0.96	0.78–1.18	0.7
Sunscreen feels bad on my skin	0.72	0.52–1.01	0.06	1.04	0.87–1.25	0.63
I don’t have access to sunscreen	1.34	0.86–2.08	0.19	1.05	0.80–1.38	0.73
A tan makes me feel attractive	1.40	0.98–2.00	0.07	1.42	1.15–1.75	0.01
Perceived Knowledge of Sun Safety						
Overall Score	0.99	0.84–1.17	0.94	1.09	0.98–1.21	0.11
Knowledge to protect the skin	1.00	0.61–1.66	0.99	1.19	0.87–1.61	0.27
Knowledge to select the appropriate sunscreen	0.78	0.52–1.18	0.24	1.01	0.78–1.30	0.96
Knowledge to check and interpret the UV index	1.12	0.83–1.51	0.46	1.22	1.01–1.48	0.04
Intentional Tanning						
Overall Score	1.50	1.18–1.91	<0.01	1.32	1.16–1.49	<0.01
Tanning bed use	1.92	0.96–3.85	0.07	1.4	0.98–2.01	0.07
Intentional outdoor tanning	1.76	1.21–2.55	<0.01	1.35	1.11–1.64	<0.01

* ORs interpreted as change for each level of response. AOR adjusted for skin sensitivity (6-point scale).

## Data Availability

The data presented in this study are available on request from the corresponding author. The data are not publicly available.
